# An Unusual, Intermediate-Sized Lesion Affecting Motor Organization in a Patient With Schizencephaly: A Case Report

**DOI:** 10.3389/fnhum.2020.00258

**Published:** 2020-07-10

**Authors:** Sung-Woon Baik, Gi-Wook Kim, Myoung-Hwan Ko, Jeoung-Hwan Seo, Yu-Hui Won, Sung-Hee Park

**Affiliations:** ^1^Department of Physical Medicine and Rehabilitation, Chonbuk National University Hospital, Jeonju, South Korea; ^2^Research Institue of Clinical Medicine of Chonbuk National University—Biomedical Research Institue of Chonbuk National University Hospital, Jeonju, South Korea; ^3^Translational Research & Clinical Trial Center for Medical Device, Chonbuk National University Hospital, Jeonju, South Korea

**Keywords:** schizencephaly, transcranial magnetic stimulation, diffusion tensor imaging, polymicrogyria, congenital brain anomalies

## Abstract

Schizencephalies are abnormal clefts of the cerebral hemispheres that result from abnormal late neuronal migration and cortical organization. In the present study, we report a different type of unusual motor organization in a patient with a schizencephalic cleft in the right hemisphere and polymicrogyria in the opposite hemisphere. Despite similar brain pathology affecting the sensorimotor cortex, motor organization differed from previously known bilateral congenital brain lesions. We conducted a transcranial magnetic stimulation (TMS) and diffusion tensor image (DTI) study to confirm the motor organization. In this case, ipsilateral corticospinal projections to the paretic hands were observed during TMS of the less affected hemisphere, along with polymicrogyria, similar to the previous study. However, a crossed corticospinal tract to the paretic hand from the more severely affected hemisphere was observed in this case-a pattern of motor organization that has yet to be reported in this patient population. Our findings indicate that motor organization after early brain injury may be affected by the interhemispheric competition of the corticospinal system and bilateral brain lesions, thereby resulting in unilateral hemiparesis.

## Introduction

Schizencephalies are abnormal clefts of the cerebral hemisphere caused by the enfolding of gray matter, extending from the cerebral wall to the pial surface of the ependyma of the underlying ventricle (Barkovich and Norman, 1988; Hong et al., 1991). Characteristically, these abnormalities affect the perisylvian region and the precentral and postcentral gyri (Hong et al., 1991). As this developmental abnormality occurs during the second trimester of pregnancy when brain plasticity is thought to be highest, schizencephaly is more likely to be associated with effective sensorimotor reorganization (Maegaki et al., 1995; Eyre et al., 2001; Staudt et al., 2002; Eyre, 2007). Cortical dysplasia can be co-occurred broadly, near the cleft, or rarely contralateral hemisphere of the cleft (Braga et al., 2018). Patients with schizencephaly have various clinical manifestations including developmental delay, motor dysfunction, neurocognitive dysfunction, and seizures (Barkovich and Kjos, 1992).

Transcranial magnetic stimulation (TMS) is an ideal noninvasive method to confirm the motor pathway characteristics (Rossini et al., 1998). It can also distinguish between a corticospinal tract and a non-corticospinal tract motor pathway based on MEP characteristic analysis (Kim et al., 2004). Ipsilateral motor evoked potentials (iMEPs) from the corticospinal tract have similar latency and amplitude to that of contralateral motor evoked potentials (cMEPs). However, ipsilateral MEPs from the non-corticospinal tract has more delayed latency and smaller amplitude than those from a contralateral tract (Kim et al., 2004). Persistence of fast-conducting ipsilateral corticospinal projections from the unaffected hemisphere-an indicator of early sensorimotor reorganization and motor compensation-has previously been demonstrated in patients with schizencephaly (Maegaki et al., 1995; Eyre et al., 2001; Staudt et al., 2002; Eyre, 2007; Son et al., 2008). However, the presence of crossed corticospinal projections from the schizencephalic hemisphere with uncrossed ipsilateral corticospinal projections from the non-schizencephalic hemisphere has yet to be reported among patients with schizencephaly.

Diffusion tensor imaging (DTI) can also offer a more accurate identification of the white matter dysgenesis and injury, than the conventional magnetic resonance imaging (MRI).

In the present report, we describe a different type of motor organization in a patient with schizencephaly accompanied by polymicrogyria. No reports have identified intermediate-sized lesions associated with the motor organization in patients with schizencephaly. Also, we report additional findings obtained *via* DTI to investigate the mechanisms underlying the pathogenesis of schizencephaly that lead to different types of motor organization.

## Case Report

A 25-year-old man admitted to our department for the management of spasticity and weakness in all extremities, with left side predominance, which was present from infancy. He was born at full term, with normal vaginal delivery, and without any perinatal complications. Physical examination for motor power by manual muscle test showed grade 4–5 in right side extremities, grade 3–4 in left upper extremity, and grade 2–3 in left lower extremity. The grip strength with JAMAR Hydraulic Hand Dynamometer (Sammons Preston, Chicago, IL, USA) for each hand was 18 kg in the right hand (normal: 53.90 ± 9.6 kg) and 9 kg in the left hand (normal: 50.00 ± 8.9 kg; Bohannon et al., 2006). In the 9-hole pegboard test, it was noted that he took 37 s for the right hand (normal: about 19 s) and 140 s for left hand (normal: about 20 s; Wang et al., 2015). There were no limitations on joint range of motion. Spasticity was checked by the modified Ashworth scale (MAS). It showed MAS I grade in the right lower extremity, MAS I+ grade in left upper extremity, and MAS II in the left lower extremity. He had delayed developmental milestones and was able to walk around 5 years of age. Also, he started speaking at age 5 and could speak two words joined together at age 8. Moderate mental retardation (IQ 45) was documented on Korean Wechsler Adult Intelligence Scale. Mirror movement was assessed by three different tasks, suggested by (Woods and Teuber, 1978), finger tapping, fist turning, and finger alternation. The score of the mirror movement was six in the right hand and four in the left hand. Non-paretic hand showed more mirror movements than the paretic hand. He had no history of epilepsy nor took drugs that may affect the cortical activity. And, he had no other medical, family, and psycho-social history.

**Figure 1:**
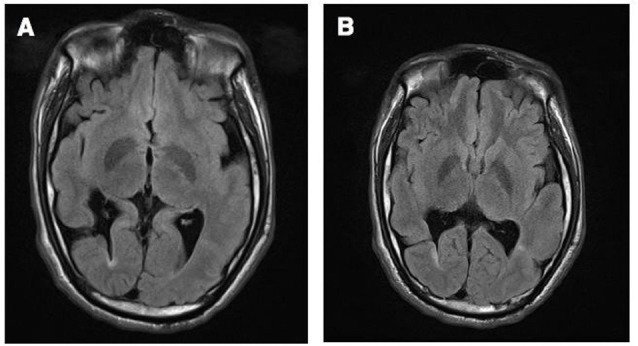
Brain magnetic resonance imaging (MRI) revealed a closed-lip schizencephalic cleft in the right hemisphere **(A)**, and non-schizencephalic focal cortical dysplasia in the left hemisphere **(B)**.

He had a bilateral congenital brain lesion, which was identified by a brain MRI, as a closed-lip schizencephalic cleft in the right hemisphere and focal cortical dysplasia in the left hemisphere (Figure 1). Additional cortical malformations were also observed. The extent and involvement of the schizencephalic cleft in the right hemisphere were greater in this case, and also cortical dysplasia in the left hemisphere was more severe than previously reported cases (Kim et al., 2004).

The TMS system utilized was a Magpro (Medtronic Inc., Skovlunde, Denmark) with a 70 mm figure-of-8 shaped coil. The recordings of iMEPs and cMEPs were made simultaneously at the bilateral first dorsal interosseous (FDI) muscles. The patient was evaluated in a relaxed sitting position. The coil was held tangentially to the scalp, with the handle angled backward and 45° away from the midline. The minimal time interval between stimuli was 10 s and the coil temperature was kept <35°C. Initially, we searched for the motor hotspot, which was the point where TMS produced the largest MEP, using a fitting cap pre-marked with sites at 1 cm spacing in the latitude-longitude coordinate system to navigate. Then, we measured the resting motor threshold (RMT), which was defined as the minimum stimulation intensity required to evoke an amplitude of >50 μV in at least 5/10 consecutive trials. We calculated the onset latency and peak-to-peak amplitude by averaging the values from four stimuli at 110% of RMT. To evaluated the central motor conduction time (CMCT), we stimulated the cervical spine (over C5-6) with a 140 mm diameter round coil whilst recording from the FDI. Stimulation was defined using the supramaximal method. CMCT was defined as the difference in latency to the FDI between motor cortical and cervical spinal cord stimulation.

**Figure 2:**
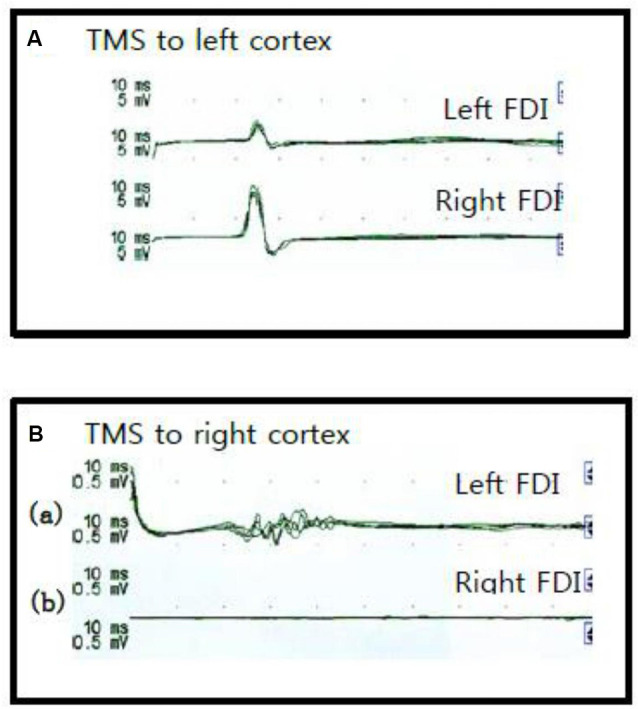
Recording of the right and left first dorsal interossei motor evoked potentials during transcranial magnetic stimulation (TMS). There was ipsilateral and contralateral first dorsal interossei response during left cortex stimulation **(A)**. Also, there was a contralateral first dorsal interossei response during the right cortex stimulation **(B)**.

Optical stimulation sites for FDI muscles were identified about 1 cm anterior and 5 cm lateral to the vertex on both hemispheres. The RMT at the optimal stimulation was 54% in the left hemisphere for the cMEPs and iMEPs. And the RMT in the right hemisphere was 90% for the cMEPs. When left hemisphere was stimulated, we observed iMEPs on patient’s paretic hand (latency, 21.9 ± 0.1 ms; amplitude, 2.1 ± 0.1 mV; duration, 12.0–14.0 ms) and cMEPs on non-paretic hand (latency, 21.2 ± 0.2 ms; amplitude, 4.2 ± 0.3 mV; duration, 14.0–16.0 ms). We also observed cMEPs on the patient’s paretic hand (latency, 23.7 ± 0.4 ms; amplitude, 153.8 ± 25.7 μV; duration, 25 ms) during right hemisphere stimulation (Figure 2). CMCT from the motor cortex to left FDI was 10.4 ms and to right, FDI was 8.1 ms. Any adverse effects of TMS such as syncope, seizure, headache, hearing problems, or changes in emotion were not reported.

In this case, cMEPs from the more severely affected hemisphere were observed, in the paretic hand. Therefore, in this case, MEPs could be elicited in the paretic hand *via* TMS of the right schizencephalic hemisphere and the opposite dysplastic hemisphere. TMS of the less affected left hemisphere elicited MEPs in the right hand as well as the paretic left hand. Polyphasic MEPs with prolonged duration were observed in the left paretic FDI when applying TMS to the schizencephalic right hemisphere.

DTIs were obtained with a head coil on a 3.0T Siemens Verio scanner (Siemens, Erlangen, Germany) using a single-shot echo-planar imaging sequence with two diffusion sensitizing gradients. To reduce the duration of the scan, the Generalized Autocalibrating Partially Parallel Acquisition (GRAPPA) technique, which is a part of the parallel imaging technique was used. Moreover, this technique produces better image quality because of the reduction in image distortion caused by the echo-planar imaging sequence. For correcting the potential image distortion, an automated image registration program was employed. The image parameters used were: echo time = 93 ms, repetition time = 7,900 ms, field of view = 230 mm^2^, matrix size = 128 × 128 reconstructed with a homodyne to 256 × 256, SENSE factor = 3, EPI = 128 and *b*-value = 1,000 s/mm^2^. We acquired 47 contiguous slices of 3.0 mm slices, parallel to the anterior commissure-posterior commissure line with no gap in 30 different diffusion directions. Anisotropy was calculated by using the orientation-independent fractional anisotropy (FA) and DTI color maps were created from FA values and the three vector elements. Vector maps were assigned colors viz, red (x, left-right), green (y, anteroposterior), and blue (z, superior-inferior) with a proportional intensity scale according to the FA. Visual assessment of white matter fibers on DTI color maps was performed.

**Figure 3:**
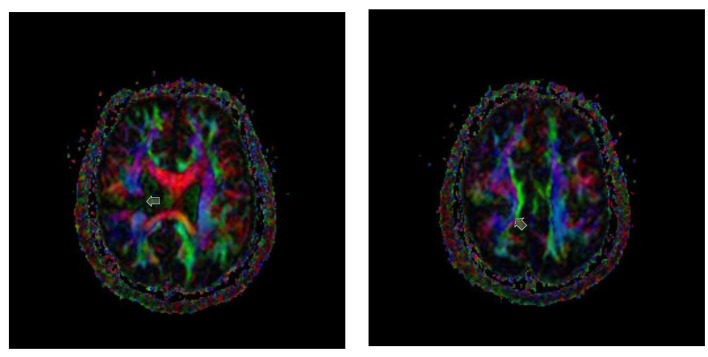
Axial color map showing right-sided cleft extension to the midline (arrow), leading to interruption of the superior corona radiate, which contained the corticospinal projections and thalamocortical fibers in blue color.

The axial color map revealed right-sided cleft extension to the midline, leading to interruption of the superior corona radiate, which contained the corticospinal projection and thalamocortical fibers. Distorted and deviated white matter bundles were also visualized around the schizencephalic cleft (Figure 3).

## Discussion

In the present case, we identified an intermediate-sized lesion affecting motor organization in the patient with schizencephaly. Previously, we reported the case of a Korean patient whose paretic hand maintained uncrossed ipsilateral corticospinal projections from the non-schizencephalic hemisphere only (Son et al., 2008). Although brain lesions and clinical manifestations were similar to the previous case, the motor organization differed from that case. In the previously reported case, TMS of the less affected left hemisphere elicited MEPs in the paretic and non-paretic hands, whereas no motor responses were elicited by TMS of the more affected schizencephalic hemisphere, even when the stimulation intensity was increased to 100% of the maximal output (Son et al., 2008). However, in this case, TMS of the less affected left hemisphere showed similarly, but there was a cMEP to the paretic hand from the more affected right hemisphere which confirmed by TMS. This has not been reported previously to our knowledge.

Mirror movements in the paretic hand considered as the sign of ipsilateral corticospinal projections from the unaffected hemisphere and may suggest favorable motor outcomes in early brain injury (Woods and Teuber, 1978). There are two types of mirror movement, physiological and pathological. The physiological mirror movement usually appears under 5 years old and the most usual reason for this movement is the deterioration of maturity of the corpus callosum (Reitz and Müller, 1998). On the other hand, pathological mirror movement usually appears at more than 5 years old and this movement is divided into congenital mirror movement and acquired mirror movement. In congenital mirror movement, iMEPs appear in both hands and iMEPs can become dominant (Kim et al., 2016). Acquired mirror movement can be seen at only one hand and cMEPs become dominant. In our case, the patient showed stronger mirror movement in the non-paretic hand during voluntary tasking of the paretic hand. This is as per TMS findings that the non-paretic hand mainly controlled by the crossed corticospinal projections from the less affected hemisphere. Also, in the paretic hand, it showed a bit weaker mirror movement than the non-paretic hand. It is because of the evoked TMS response of the crossed corticospinal projections from the schizencephalic hemisphere.

Several TMS studies have investigated motor organization in patients with schizencephaly (Maegaki et al., 1995; Kim et al., 2004; Son et al., 2008). In these reports, the motor organization of the paretic hand was associated with large-sized lesions, and the paretic hand only received ipsilateral corticospinal projections from the unaffected hemisphere, as observed in the previous case (Son et al., 2008). However, previous authors have suggested that corticospinal pathways may originate from the schizencephalic hemisphere, as research has indicated that intraoperative electrical stimulation elicits motor responses from areas of the cortex lining the schizencephalic clefts (Granata et al., 1996; Staudt et al., 2002). Also, some authors have reported that functional MRI activation can be observed in the dysgenic cortex lining the schizencephalic clefts, even without TMS evidence of corticospinal projections originating from these sites (Staudt et al., 2002).

The prolonged duration of contralateral MEPs from the affected hemisphere is found at normal maturation of corticospinal tract projection or found at the pathological situation of the corticospinal tract (Nezu et al., 1996; Nezua et al., 1997). Also, delayed latency of contralateral MEPs from the affected hemisphere is a characteristic of the reorganization of central motor pathways after the ischemic change (Hömberg et al., 1991). There are several reports that polyphasic MEPs is caused by pathological alternations of the corticospinal pathways due to demyelination and remyelination (Firmin et al., 2012; Min et al., 2012).

In this case, contralateral MEPs in the paretic hand were polyphasic, implying the delayed electrophysiological maturation or the pathologic condition of the corticospinal motor pathways. In contrast, the ipsilateral MEP in the paretic hand showed very similar patterns of contralateral one in the nonparetic hand. These findings may suspect that ipsilateral MEPs from paretic hand and contralateral MEPs from non-paretic hand are from the same origin.

Although brain pathology was similar to the previous case, differences in the extent, location, and severity of brain lesions may have led to different types of motor organization. The unaffected hemisphere can maintain ipsilateral corticospinal projections to the paretic hand, and this can frequently be observed in patients with congenital brain lesions. However, few reports have indicated that the less affected hemisphere can compensate for motor function in patients with bilateral congenital brain lesions, as observed in this case. Moreover, the less affected hemisphere preserved fast-conducting ipsilateral corticospinal projections to the paretic hand, similar to findings observed for the unaffected hemisphere in patients with unilateral brain lesions. To our knowledge, the present study is the first to demonstrate these findings.

We believe that these differences in the organization may have been due to interhemispheric competition of the corticospinal system, based on lesion severity. When the activity of neurons in one sensorimotor cortex is inhibited during development, it fails to maintain contralateral terminations and is taken over by increased ipsilateral terminations from the normally active hemisphere (Eyre et al., 2001). In contrast, when both sensorimotor cortices are inhibited, an essentially normal pattern of ipsilateral and contralateral terminations is maintained from both hemispheres. Such competition is likely to occur in this case. Bilateral activity-dependent interactions of the corticospinal system may determine the development of crossed corticospinal projections from the more affected hemisphere, even when the less affected hemisphere maintains ipsilateral corticospinal projections to the paretic hand. In this case, the less affected hemisphere exhibited relatively greater motor activation, which may have reduced activity in the affected hemisphere such that it could not develop corticospinal projections.

One can argue that, in this case, schizencephalic cleft may not have been severe enough to disrupt corticospinal projections, allowing the schizencephalic hemisphere to maintain corticospinal motor projections. However, axial color FA from DTI indicated that the schizencephalic cleft may also have affected the superior corona radiata. Therefore, this hypothesis is unlikely.

In the present study, we identified different patterns of motor organization in a patient with unilateral, closed-lip schizencephaly and contralateral cortical dysplasia, despite the very similar extent and location of cortical malformations and their common brain pathology from that of the previously reported case. Although, we could only qualitatively assess the locations and patterns of the malformations *via* radiological analysis, differences in the extent and severity of brain lesions between the two cases may have led to differences in motor organization. However, further studies involving larger sample sizes are required to verify this hypothesis. Our findings demonstrate that interhemispheric differences in the severity and extent of malformations may be an important factor for determining motor organization in patients with early brain lesions. Moreover, the extent of brain injury during early embryonic development may be affected by the interhemispheric competition of the cortical system, thereby resulting in different patterns of motor organization.

## Data Availability Statement

All datasets generated for this study are included in the article.

## Ethics Statement

The studies involving human participants were reviewed and approved by Chonbuk National University Hospital Institutional Review Board. Written informed consent for participation was not required for this study in accordance with the national legislation and the institutional requirements. Written informed consent was obtained from the patient prior to the publication of this case report and accompanying images.

## Author Contributions

S-HP: conceptualization. S-WB, G-WK, Y-HW, M-HK, and J-HS: methodology. S-WB and S-HP: formal analysis. S-WB, G-WK, Y-HW, M-HK, J-HS, and S-HP: project administration and writing—review and editing. S-WB and S-HP: visualizatioin. S-WB and S-HP: writing—original draft. All authors: approval of final manuscript.

## Conflict of Interest

The authors declare that the research was conducted in the absence of any commercial or financial relationships that could be construed as a potential conflict of interest.
